# Profile of the urinary excretion of prednisolone and its metabolites in finishing bulls and cows treated with a therapeutic schedule

**DOI:** 10.1186/s12917-014-0237-0

**Published:** 2014-09-30

**Authors:** Carlo Nebbia, Pierluigi Capra, Marta Leporati, Flavia Girolami, Gandolfo Barbarino, Stefano Gatto, Marco Vincenti

**Affiliations:** Dipartimento di Scienze Veterinarie, Università di Torino, Largo Paolo Braccini 2, Grugliasco (Torino), 10095 Italy; Istituto Zooprofilattico Sperimentale del Piemonte, Liguria e Valle d’Aosta, Bologna 148, 10154 Torino, Italy; Centro Regionale Antidoping e di Tossicologia “Alessandro Bertinaria”, regione Gonzole 10/1, Orbassano (Torino), 10043 Italy; Regione Piemonte, Assessorato Tutela della Salute e Sanità, Settore Prevenzione e Veterinaria, Via Lagrange 24, Torino, 10123 Italy; ASL TO3, Servizio Veterinario, Via Poirino, 9, Pinerolo (TO), 10064 Italy; Dipartimento di Chimica, Università degli Studi di Torino, via Pietro Giuria 7, Torino, 10125 Italy

**Keywords:** Prednisolone acetate, Therapeutic treatment, Finishing bulls, Cows, LC/MS-MS, Prednisone, 20β-dihydroprednisolone, Urinary excretion

## Abstract

**Background:**

Prednisolone was one of the first glucocorticoids to be synthesised, but it is still widely applied to cattle. Illegal uses of prednisolone include its uses for masking a number of diseases before animal sale and, at lower dosages for extended periods of time, for the improvement of feed efficiency and carcass characteristics. Since occasional presence of prednisolone has been detected at trace level in urine samples from untreated cattle, the Italian Ministry of Health introduced a provisional limit of 5 ng/mL to avoid false non-compliances. However, this limit proved ineffective in disclosing prednisolone misuse as a growth-promoter. In the present study, prednisolone acetate was administered to finishing bulls and cows according to a therapeutic protocol (2 × 0.4-0.5 mg/kg bw i.m. at 48 h interval) to further verify the practical impact of this cut-off limit and develop sound strategies to distinguish between exogenous administration and endogenous production. Urinary prednisolone, prednisone, 20β-dihydroprednisolone, 20α-dihydroprednisolone, 20β-dihydroprednisone, 6β-hydroxyprednisolone, cortisol, and cortisone were determined using a validated LC/MS-MS method.

**Results:**

The urinary excretion profile showed the simultaneous presence of prednisolone, 20β-dihydroprednisolone, and prednisone, the latter at lower concentrations, up to 33 days after the first dosing. Higher analyte levels were detected in bulls even after correction for dilution in the urine. Prednisolone concentrations below 5 ng/ml were determined in half of the samples collected at 19 days, and in all the samples obtained 26 and 33 days after the first administration. No measurable concentrations of prednisolone or its metabolites were found in the samples collected before the treatment, while cortisol and cortisone levels lower than the respective LOQs were observed upon treatment.

**Conclusions:**

The present study confirms the criticism of the coarse quantitative approach currently adopted to ascertain illegal prednisolone administration in cattle. As previously shown for growth-promoting treatments of meat cattle, the simultaneous determination of urinary prednisolone, prednisone, 20β-dihydroprednisolone, along with cortisol and cortisone, may represent a more reliable approach to confirm the exogenous origin of prednisolone. Such a strategy would facilitate unequivocal detection of animals treated with prednisolone acetate using a therapeutical protocol, even 3 to 4 weeks after the treatment.

**Electronic supplementary material:**

The online version of this article (doi:10.1186/s12917-014-0237-0) contains supplementary material, which is available to authorized users.

## Background

Synthetic glucocorticoids are extensively employed in cattle therapeutic treatments due to their well recognized anti-inflammatory, anti-allergic, and metabolic properties. They are used in the treatment of primary ketosis, disorders of the musculoskeletal system, allergic reactions, skin diseases, and shock [1]. Despite its relatively low glucocorticoid potency in comparison with synthetic fluorinated derivatives (e.g. dexamethasone, betamethasone, flumethasone or isoflupredone), prednisolone is still widely used in cattle, including dairy cows, particularly for its effectiveness in the treatment of mastitis, in conjunction with antimicrobial drugs [2,3].

Besides therapeutic applications, synthetic glucocorticoids (including prednisolone) are being increasingly used illegally as growth promoters in veal calves, finishing bulls and possibly old cows at the end of their productive cycle, giving rise to a relatively high incidence of non compliances [4]. Significant live weight increase, coupled with an improvement of both feed conversion and carcass quality traits [5,6], have been reported in treated animals, as the result of several pharmacological mechanisms, including gene modulation at muscle level [7]; in this case, the oral route is usually preferred, and involves the administration of low dosages for an extended period of time. As for most glucocorticoids, growth-promoting effects have been demonstrated for prednisolone, orally administered as its acetate ester in beef cattle (15–30 mg *per capita*/day) for 30–35 days [8,9]. In addition, prednisolone is also likely to be administered to hide traumatic pathologies just before the animal sale, or to speed up the drying-off period in dairy cows, a well-known side effect of glucocorticoids, which is linked to both the inhibition of peripheral glucose utilization and the effects on water and electrolyte balance brought about by such drugs [10,11]. In either case, the treatment is seldom recorded officially and, consequently, appropriate withdrawal periods are rarely applied.

To protect the consumer’s health, Maximum Residue Limits for prednisolone have been set for bovine meat, liver, kidney, fat, and milk [12]. In the absence of an official record of the treatment, the occurrence of measurable levels of the drug in urine samples collected at the farm or the slaughterhouse is considered to reflect the misuse or abuse of this glucocorticoid [13].

Recently, evidence has been provided that veal calves and cows, under heavy stress conditions, would produce endogenous prednisolone, resulting in the presence of trace amounts of the glucocorticoid in the urine together with remarkable levels of cortisol and cortisone [14]. The structural affinity between cortisol and prednisolone has raised the hypothesis that the latter could originate from the former. Moreover, the occurrence of prednisolone was demonstrated in bovine adrenals and other organs from untreated cattle [15,16]. However, neither the tissue(s) nor the metabolic pathway(s) involved in the alleged endogenous synthesis of prednisolone has been clarified yet.

To avoid false non-compliances, a provisional limit of 5.0 ng/mL for prednisolone in cattle urine was established in 2012 by the Italian Ministry of Health, in agreement with a suggestion of the European Union Reference Laboratory [17]. However, previous studies revealed that the oral dosing of beef cattle with prednisolone acetate according to a growth-promoting protocol never resulted in prednisolone concentrations higher than 1 ng/mL in urine specimens collected during the treatment [8,18]. Therefore, the current 5.0 ng/mL limit is likely to generate false negative results, with the potential for the exposure of consumers to harmful residues. On the other hand, only scant data are presently available to assess the practical impact of the provisional prednisolone urinary limit of 5.0 ng/mL in cattle undergoing a pharmacological treatment with the glucocorticoid [19].

The main aims of the present study were to characterise the urinary excretion profile of prednisolone after intramuscular (i.m.) administration to healthy finishing bulls and cows using a therapeutic schedule and the applicability of the 5.0 ng/mL urinary limit to such animals. Moreover, in agreement with the approach followed for bullocks administered with a growth-promoting protocol [18], the urinary concentrations of cortisol, cortisone, prednisone, and of further prednisolone metabolites were also determined with the aim to develop an appropriate interpretation strategy to distinguish the urinary presence of exogenous prednisolone from that related to the alleged endogenous synthesis .

## Methods

### Animals, treatments, and sample collection

**Figure 1:**
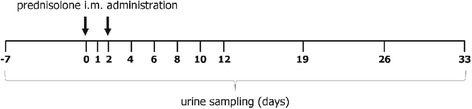
**Treatment schedule and urine collection.** Finishing bulls and cows were treated i.m. according to a therapeutical protocol (2 × 0.4-0.5 mg prednisolone acetate/kg body weight at 48 h interval). Urine was collected at the indicated timepoints up to 33 days after the first administration.

The study was conducted on eight Charolais finishing bulls (average weight of about 400 kg) and six Friesian non-lactating cows at the end of their productive cycle (average weight of about 500 kg). The experimental plan was designed according to the guidelines of the Italian law for care and use of experimental animals [20], and the study was approved by the Ministry of Health and the local Committee for Animal Welfare. The animals were clinically healthy and bred with a standard diet. After an acclimatization period of one week (Figure 1), they were subjected to a first urine sampling (T = −7) and after a further week to a second one (T = 0) just before being i.m. treated with prednisolone acetate at the maximum recommended dosage for cattle. To mimic conditions usually applied by the practitioners, all individuals received 14 mL of a commercial preparation (Novosterol®, Ceva Vetem SPA, Italy) containing 15 mg prednisolone acetate/mL suspension, corresponding to 210 mg active principle/head (approximately 0.5 or 0.4 mg/kg body weight, for bulls and cows, respectively). A second dosing was performed after 2 days (T = 2). Urine sampling (spontaneous micturition) was also performed 1, 2, 3, 4, 6, 8, 10, 12, 19, 26, and 33 days after the first treatment, respectively, usually late in the morning (11 a.m.). After collection, urine specimens were immediately stored at −20°C pending their analysis.

### Chemicals and reagents

Diethylether and acetonitrile were supplied by Sigma–Aldrich (St. Louis, MO, USA). Sodium hydroxide and hydrochloric acid were purchased from Carlo Erba Reagenti (Milan, Italy). β-glucuronidase/aryl-sulfatase was from Roche Diagnostics (Mannheim, Germany). 20α-dihydroprednisolone, 20β-dihydroprednisolone, 6β-hydroxyprednisolone and 20β-dihydroprednisone were supplied by Steraloids (Newport, RI, USA). Prednisone, prednisolone, and cortisol were purchased from Sigma Aldrich (Milan, Italy). Triamcinolone acetonide-d6 (internal standard (IS)) was from RIVM (Bilthoven, The Netherlands). Stock standard solutions of all the analytes were prepared in acetonitrile at a concentration of 1 mg/mL and stored at −20°C in the dark.

### Sample preparation and instrumental analysis

The analytical method adopted for the determination of corticosteroids and metabolites, along with the figures of merit resulting from the validation process were reported in previous studies [18,21]. Briefly, urine samples were subjected to a liquid/liquid extraction at pH 8.5–9.5 with diethylether after β-glucuronidase/arylsulfatase deconjugation. After centrifugation, the supernatant organic phase was transferred into a 10-mL glass tube and evaporated to dryness under nitrogen at 40°C. The residue was re-dissolved in 50 μL of H_2_O/CH_3_CN (70/30) mixture and transferred into an analytical vial for the instrumental analysis, performed with a LC-MS/MS apparatus. An Agilent 1100 series liquid chromatograph (Agilent Technologies, Palo Alto, CA, USA), was interfaced to an Applied Biosystems API 4000 triple quadrupole mass spectrometer (Applied Biosystems Sciex, Ontario, Canada), operating in atmospheric pressure chemical ionisation positive ion mode.

The essential parameters and figures of merit of the latter analytical method are reported in Table 1.

**Table 1:** **Essential parameters and figures-of-merit of the analytical method adopted for determination of cortisol, cortisone, prednisolone and some of its metabolites**

**Analyte**	**Retention time (min)**	**Precursor ion m/z**	**Product ions**	**CCα (ng/mL)**	**LOD (ng/mL)**
6β-hydroxyprednisolone	4,3	377.3	**377.3**➔**341.2 Q**	--	0.41
			377.3➔323.2		
			377.3➔359.3		
20α-dihydroprednisolone	10.5	363.3	**363.3**➔**267.4 Q**	--	0.36
			363.3➔291.3		
			363.3➔309.3		
20β-dihydroprednisolone	11.6	363.3	**363.3**➔**345.2 Q**	--	0.35
			363.3➔267.3		
			363.3➔291.3		
20α-dihydroprednisone	12.3	361.3	**361.3**➔**153.2 Q**	--	0.42
			361.3➔297.3		
			361.3➔313.2		
Prednisolone	17.1	361.3	**361.3**➔**279.2 Q**	0.67	0.05
			361.3➔265.2		
			361.3➔223.2		
Prednisone	17.9	359.3	**359.3**➔**313.2 Q**	0.66	0.05
			359.3➔295.2		
			359.3➔267.2		
Cortisol	17,7	363.2	**363.2**➔**121.3 Q**	--	0.1
			363.2➔147.3		
			363.2➔309.4		
Cortisone	18.9	361.2	**361.2**➔**163.4 Q**	--	0.1
			361.2➔121.3		
			361.2➔105.3		
Triamcinolone Acetonide D6	27.1	441.4	**441.4**➔**421.3 Q**	--	--
			441.4➔403.4		

*Q: Quantifier transition.

**CCα: Decision Limit.

***LOD: Limit of Detection.

Validation of the methods was not performed according to the Commission Decision 2002/657/EC for all the analytes. Since only prednisolone and prednisone are included in official monitoring plans, CCα has been determined only for these two analytes. For the other prednisolone/prednisone metabolites, and for cortisol and cortisone as well (both included into the method in a second time) a simpler validation approach was carried out, with LOD calculation only.

All urine samples were analyzed with the method initially developed for prednisolone, prednisone, cortisol, and cortisone [21]. Later on, after the new analytical method for the entire set of metabolites was developed and validated [18], the residual urine samples were analyzed once more for the presence of metabolites and creatinine (see 2.4). The stable concentrations of prednisolone and prednisone proved that the residual samples remained unchanged during storage at −20°C and despite the single freeze-and-thaw cycles intervened. For the reasons outlined above, a complete set of samples was no longer available; the residual urine volumes allowed the analysis to be performed on the majority of bull and cow specimens collected 0, 1, 3, 19, and 33 days after the first administration. Scattered data at different time points on a reduced set of individuals were also available, but they did not change the interpretation of the results, so they have not been reported. All the raw data concerning prednisolone, prednisone, and the measured metabolites, upon which the following discussion is based, are available as an additional file (Table T1-T6 in “Additional file 1”).

### Determination of creatinine

Creatinine is an endogenous by-product of muscle activity, which is produced with limited intra-day variation in a given individual and cleared at a constant rate by the kidney. Therefore, it has been long used to normalize urinary values of certain analytes (e.g. cortisol) to account for individual hydration and time since last urination [22,23]

Urinary creatinine was measured using two methods, a creatinine assay by ARCHITECT C8000 System (Abbott, Abbott Park, IL, USA) and a LC-MS/MS method [24].

The creatinine assay is based upon the reaction between creatinine and sodium picrate to form a creatinine-picrate complex. The rate of increase in absorbance at 500 nm due to the formation of this complex is directly proportional to the concentration of creatinine in the sample. Since the creatinine kit is proposed for the quantitation of creatinine in human serum, plasma or urine and not for bovine urine, ten samples (out of 56, tested with the creatinine kit) were analysed also by means of LC-MS/MS. No substantial differences were found between the two sets of results.

The LC-MS/MS apparatus was the same employed for prednisolone and its metabolites determination, but with an electrospray ionization source (ESI), operating in positive ion mode. The MS parameters were set as follows: curtain gas: 10 psi; ion source gas-1: 40 psi; ion source gas-2: 40 psi; ion spray voltage: 5000 V; probe temperature:350 C; declustering potential: 31 V; entrance potential: 10 V. Three transitions of parent ion to product ion were considered (114.0➔ 44.2; 114.0➔ 86.1; 114.0➔ 72.0). Their optimal values of collision energy and collision cell exit potential were: CE = 29 V and CXP = 6 V for product ion 44.2; CE = 16 V and CXP = 15 V for product ion 86.1; CE = 22 V and CXP = 12 V for product ion 72.0.

The LC system was equipped with a Phenomenex Luna HILIC column (100 × 4.6 mm, 3 m) and a Phenomenex SecurityGuard 4.0 mm × 2.0 mm pre-column. The chromatographic run was carried out using a binary mobile phase of 0.1% HCOOH in highly purified water (A) and acetonitrile (B), under the following program: isocratic condition of 50% acetonitrile for 2 min; linear gradient from 50% to 10% acetonitrile in 5 min; isocratic with 10% acetonitrile for 1 min; linear gradient from 10% to 50% acetonitrile in 1 min; total run time 15 min. The injection volume was 10 μL and the flow rate was 0.4 mL/min.

### Statistics

Where appropriate, statistical differences were evaluated by Student’s t-test with GraphPad InStat version 3.00 (GraphPad Software, San Diego, CA, USA) at a 5% significance level (P < 0.05).

## Results

### Kinetics of prednisolone and prednisone urinary excretion in finishing bulls and cows

**Figure 2:**
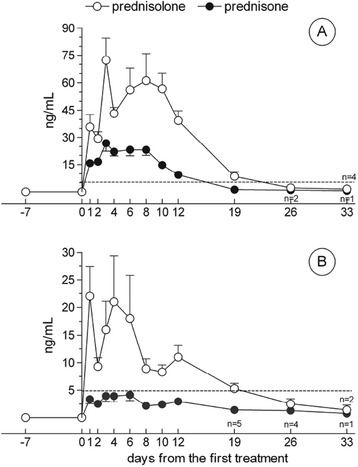
**Urinary excretion profile of prednisolone and prednisone in finishing bulls and cows i.m. treated with prednisolone acetate.** Finishing bulls and cows were treated i.m. with the glucocorticoid with a therapeutical dosage at T = 0 and T = 2. **A)** finishing bulls (n = 8); **B)** cows (n = 6). Figures under or over the graph symbols at T = 26 and T = 33 indicate the number of animals exhibiting measurable values (>CCα). Further information may be found in the supplementary files.

Figure 2 displays the profile of prednisolone and prednisone urinary excretion in treated bulls and cows. Neither compound could be detected in any urine specimen collected before drug dosing in cattle of either sex.

In finishing bulls (Figure 2A), the urinary prednisolone concentration peaked 24 hours after the second administration (T = 3) with an average level of 72 ng/mL, showing a progressive decline thereafter, with average concentrations between 40 and 60 ng/mL until T = 12, i.e. 10 days after the second injection. Measurable prednisolone levels (>CCα) were detected in all animals at T = 19 and T = 26 and in four out of eight individuals at T = 33, corresponding to 31 days after the second administration. For four out of eight urine samples collected at T = 19, and all those obtained at both T = 26 and T = 33, prednisolone concentration was below 5 ng/mL. Prednisone was already detectable 24 hours after the first administration (T = 1); its concentration peaked at T = 3 (around 27 ng/mL) exhibiting a slow decrease thereafter, corresponding to average concentrations of about 9 ng/mL at T = 12 and 1 ng/mL at T = 19. Levels of prednisone lower than 1 ng/mL but higher than the CCα could be detected at T = 26 and T = 33 in two and one individuals, respectively.

For cows (Figure 2B), the highest prednisolone concentrations were observed in urine samples collected 24 hours (T = 1) and 96 hours (T = 4) after the first dosing, with average values of 22 and 21 ng/mL, respectively. A slow decline was subsequently observed, with concentrations ranging between 9 and 11 ng/mL; at T = 19 and T = 26, three and five out of six animals, respectively, displayed urinary concentrations above the CCα, but lower than 5 ng/mL. Thirty-three days after the first administration, low but still measurable amounts of the drug could be detected in two urine samples. Prednisone was already detectable 24 hr after the first administration, at concentrations that remained within a constant range (between approximately 2 to 4 ng/mL) throughout most of the collection period. At T = 26 and T = 33 prednisone concentrations around 1 ng/mL were measured for four and one animals, respectively.

### Urinary cortisol, cortisone, and metabolites of prednisolone and prednisone in prednisolone-treated finishing bulls and cows

The presence of the main prednisolone and prednisone metabolites, plus cortisol and cortisone was investigated in urine samples collected 0, 1, 3, 19, and 33 days after the first drug treatment, respectively. In both finishing bulls and cows, 20β-dihydroprednisolone, 20α-dihydroprednisolone, 20β-dihydroprednisone, 6β-hydroxyprednisolone, and cortisone were not detected (<LOD) in any urine sample collected before the first administration (T = 0). Cortisone remained undetectable (<LOD) in any specimen collected from bulls and cows after treatment (T = 1, T = 19, T = 33). Cortisol concentration was below the LOQ value in all urine samples collected from cows, whereas it could be determined in urine samples collected from five finishing bulls before any treatment (T = 0) at low ppb level (1.17 ng/mL, 0.92 ng/mL, 0.63 ng/mL, 1.26 ng/mL and 3.44 ng/mL, for animals A, B, C, D and H, respectively) and in samples from three individuals collected 31 days after the first administration (T = 33; 1.14 ng/mL, 0.90 ng/mL and 1.23 ng/mL, for animals A, C and D, respectively).

**Figure 3:**
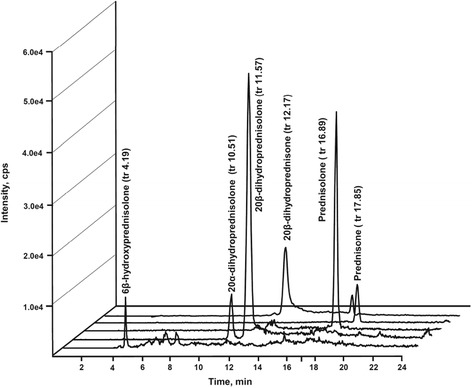
**Chromatographic profiles of a sample (from bull A) collected 1 day after the first prednisolone administration using a therapeutic schedule.** Unmodified prednisolone and all the five metabolites are present at noticeable concentrations, ranging from 7,59 ng/mL (6β-hydroxyprednisolone) to 63,5 ng/mL (20β-dihydroprednisolone).

Figure 3 represents a typical tridimensional chromatographic profiling of the metabolites detected in urine samples from a bull (Bull A) 24 hours after the first i.m. dosing with prednisolone.

**Figure 4:**
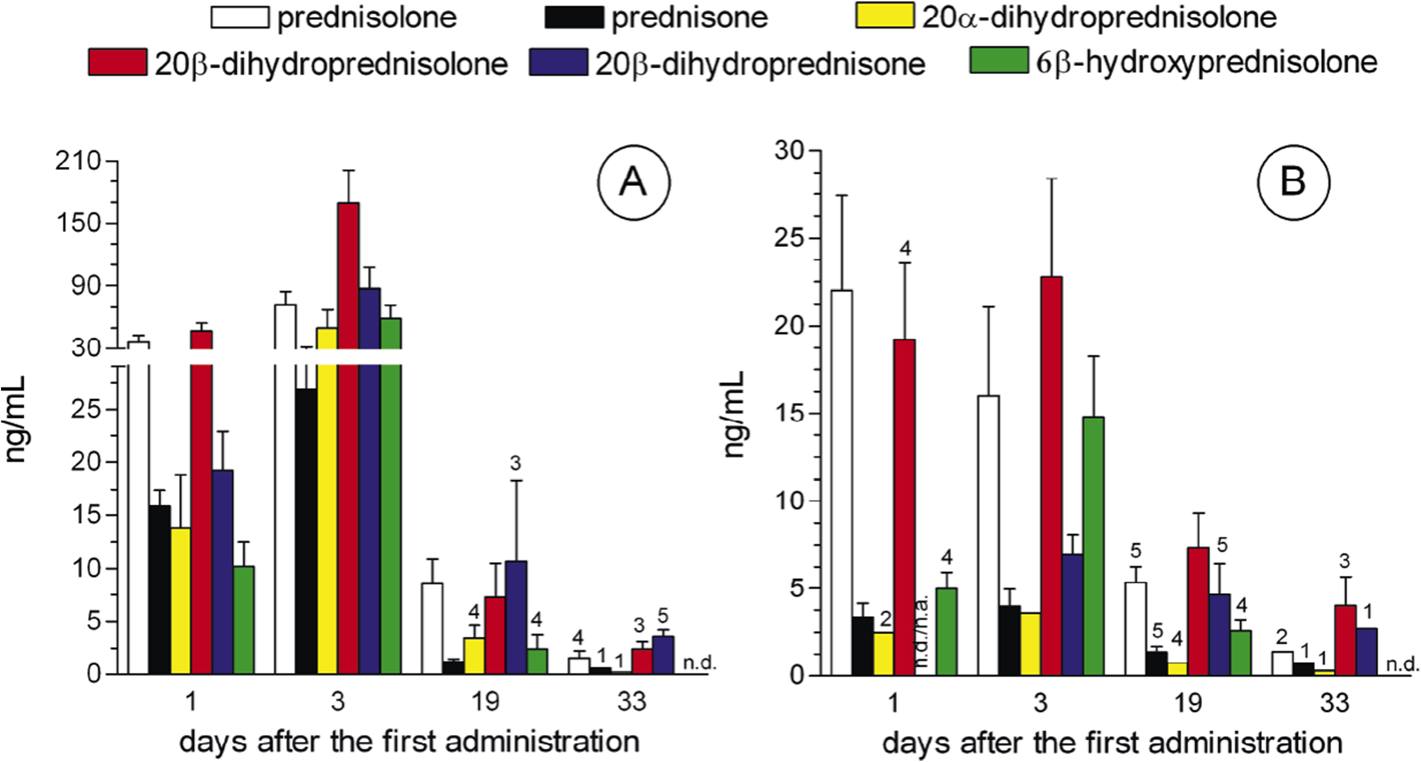
**Urinary excretion profile of prednisolone, prednisone and selected metabolites in finishing bulls and cows i.m. treated with prednisolone acetate.** Finishing bulls and cows were treated i.m. with the glucocorticoid with a therapeutical dosage. **A)** finishing bulls (n = 8); **B)** cows (n = 6). Figures over the bar symbols indicate the number of animals exhibiting measurable values (>CCα or > LOQ). n.d. = not detectable; n.a = not available. Further information may be found in the supplementary files.

Measurable amounts (>LOQ) of 20β-dihydroprednisolone, 20α-dihydroprednisolone, 20β-dihydroprednisone, and 6β-hydroxyprednisolone were detected in all urine samples from finishing bulls (Figure 4A), collected both 1 and 3 days after the first prednisolone treatment, while at T = 19 (i.e. 19 days after the first dosing), only 20β-dihydroprednisolone reached measurable levels in urine samples from all individuals. At T = 33, 20β-dihydroprednisolone and 20β-dihydroprednisone levels higher than LOQ still occurred in four and five individuals, respectively. In general, average concentrations of 20β-dihydroprednisolone (at all timepoints) and further metabolites (whenever detectable) were of the same order of magnitude as the parent drug or even higher (e.g. 20β-dihydroprednisolone at T = 3 and 20β-dihydroprednisone at T = 19 and T = 33).

In the urine samples obtained from cows (Figure 4B), 20β-dihydroprednisolone was the only metabolite that could be found in all animals at T = 1, T = 3, and T = 19, and in three out of six individuals at T = 33, always reaching concentrations similar to those of the parent drug. In general, 20α-dihydroprednisolone, 20β-dihydroprednisone, and 6β-hydroxyprednisolone exhibited measurable values shortly after drug administration (T = 1, T = 3), while in samples collected after 19 and 33 days periods, these metabolites could be detected only in few animals.

### Urinary creatinine in finishing bulls and cows

Creatinine levels in urine samples collected at T = 0, T = 3, T = 19, and T = 33 are reported in Table 2. While higher creatinine average values were recorded for bulls with respect to cows, statistically significant differences occurred at T = 3 and T = 19 only.

**Table 2:** **Urinary creatinine levels (mg/mL) at different timepoints in finishing bulls and cows treated intramuscularly with prednisolone acetate (therapeutical schedule)**

**Time (days after the first treatment)**	**Finishing bulls**	**Cows**	**Ratio**
0	1.4 ± 0.28^a^	0.93 ± 0.22^a^	1.50
3	2.0 ± 0.21^a^	0.54 ± 0.18^b^	3.70
19	1.5 ± 0.13^a^	0.86 ± 0.24^b^	1.74
33	1.3 ± 0.27^a^	0.73 ± 0.16^a^	1.78

Values represent mean ± SEM; rows with different superscripts (^a^ or ^b^) are statistically significant different (P < 0.05).

## Discussion

Despite the extensive legal use of prednisolone in bovine clinical practice and the documented potential for its abuse and misuse, only scant information is available concerning the excretion profile of this glucocorticoid in cattle urine, which is the matrix of choice for the official control in living animals in the large majority of the EU Member States [25]. The only available study was carried out in an international interlaboratory comparison test [19]: an average value of 1.58 ng/mL was reported in urine specimens from two “bovines” (140–150 kg) collected 24 hours after a single i.m. administration of 30–32 mg “prednisolone” (unspecified nature), equivalent to 0.2 mg/kg b.w.. In the present study, higher urinary prednisolone concentrations were found at about the same timepoint in bulls and cows administered with 0.4-0.5 mg/kg b.w. prednisolone dosages, corresponding to a typical therapeutical schedule for adult cattle (2 × 210 mg prednisolone acetate/head at a 24 hr interval). The different ages, dosages and administration protocols make it impossible any close comparison.

Besides urine, another matrix that can also be considered for surveillance purposes is hair. Thanks to bioaccumulation of many analytes, including steroids, the hair matrix allows their prolonged detectability. It has been used in forensic analysis and may be a matrix of choice also for long-term corticosteroid misuse determination in cattle [26,27]. However, it is not considered an official matrix for corticosteroids in Italy, and no information is available concerning the accumulation of prednisolone in cattle hair.

In the present investigation, the urinary excretion profile of prednisolone, i.m. injected to finishing bulls and cows, was characterized by a peak in the 24–48 hr period after each administration, followed by a relatively slow decline, with measurable levels of the drug in samples collected 26 days (all individuals) and even 33 days (4 bulls and 3 cows) after the first administration. These results are consistent with the slow absorption half-life (48 h), the long persistence of measurable levels in serum, and the prolonged glucocorticoid action already reported in cows administered i.m. with a suspension of prednisolone acetate at a comparable dosage [28,29]. For comparison, it is worth noting that the i.m. administration of dexamethasone sodium phosphate to finishing bulls using a therapeutic schedule (60 μg/kg b.w./day for 3 consecutive days) resulted in rapid urinary excretion, so that no detectable concentrations of the drug could be found as early as four days from the first injection [30].

Among synthetic glucocorticoids, prednisolone has the unique property to bind specifically and with high affinity to plasma corticosteroid binding globulin [31]. This and other factors may explain the sharp differences in the urinary excretion profiles between prednisolone acetate and dexamethasone sodium phosphate. While acetate esters are known to be hydrolyzed more slowly than phosphates [32], fluorinated glucocorticoids such as dexamethasone are quite refractory to oxidative, reductive and conjugative biotransformations in cattle and other ruminants [30,33,34] , so that they are mainly excreted unmodified in the urines. This is not the case for prednisolone: several studies indicate that in mammalian species including cattle [35,36], 11β-hydroxysteroid-dehydrogenases (11β-HSD) mediate the interconversion of glucocorticoids usually favouring the more active 11-hydroxy-compound over the 11-keto-metabolite. Accordingly, our investigation confirms the rapid appearance and persistence of the reduced metabolite prednisone in the bovine urine with average concentrations 2− to 7 − fold lower than prednisolone throughout the monitoring time. It is not known to what extent the interconversion of prednisolone and prednisone may contribute to the slow excretion of the parent drug and its precursor/metabolite.

In addition to prednisone, four further metabolites, namely 20β-dihydroprednisolone, 20α-dihydroprednisolone, 20β-dihydroprednisone, and 6β-hydroxyprednisolone, had been previously identified in the urine specimens collected from a bullock treated i.m. with prednisolone acetate at the same dosage used in this trial [18]. A similar metabolic profile had been reported in humans, where the 20α- and the 20β-reduced derivatives were allegedly generated by two hydroxysteroid dehydrogenases, namely 20α-hydroxysteroid dehydrogenase and 3α,20β-hydroxysteroid dehydrogenase [37], while the hydroxylated metabolite was synthesized by cytochrome P450 3A4 [38]. In the study mentioned above [18], finishing bulls, treated according to a growth-promoting protocol, exhibited urinary levels of 20β-dihydroprednisolone largely exceeding those of the parent drug, while none of the remaining metabolites could be detected. In the present study, finishing bulls and cows dosed with a pharmacological schedule exhibited a sharply different excretion profile in which prednisolone, prednisone, and 20β-dihydroprednisolone were consistently recorded at similar concentrations for all animals during and immediately after the treatment, as well as 19 days after the first administration. In addition, 20α-dihydroprednisolone, 20β-dihydroprednisone, and 6β-hydroxyprednisolone were detected in the urine samples collected from a limited number of animals at 19 and 33 days after the first administration, although their concentrations were lower than those of the parent drug and its major metabolites.

Data from this study indicate that the average urinary concentration of both prednisolone (1.5− to 7 − fold) and prednisone (1− to 10 − fold) was consistently higher for finishing bulls than for cows at timepoints up to 19 days after the first administration. A similar trend was also observed for the other metabolites. Such a difference may be partly explained by the slightly higher drug dosage received by the bulls with respect to their body weight (0.5 vs. 0.4 mg/kg bw) and the larger dilution of cow urines, as inferred from their lower creatinine levels (1.5− to 3.7 − fold, at the same timepoint). However, other factors may have also played a role, including gender differences in the basal expression and/or glucocorticoid-mediated modulation of 11β-HSD [39] or other enzymes (e.g. CYP3A) involved in the generation of the examined prednisolone metabolites [40,41].

As mentioned before, the Italian Ministry of Health has recently enacted a provisional limit of 5 ng/mL for urinary prednisolone in bovine species to account for any possible “endogenous” synthesis. Such a high limit already prevented the disclosure of prednisolone abuse as a growth-promoter in beef cattle, since the prednisolone urinary concentration remained largely below the cut-off limit for all treated animals throughout the whole experimental period [8], thus generating “false compliances” to the law requirements. According to data obtained in the present study, the therapeutic administration of prednisolone acetate to cattle would not result in “false compliances” only for urine samples collected from bulls and cows close to dosing or in the 10–12 days thereafter. Indeed, the application of the 5 ng/mL limit would have classified as compliant about half of the samples collected at T = 19 and all samples obtained at both T = 26 and T = 33. Even more striking is the case of urine samples T = 26 and T = 33 of a cow (identified with “C”) that contain prednisolone concentrations below 5 ng/mL (4.67 ng/mL and 2.26 ng/mL, respectively) and prednisone concentrations above the CCα (2.19 ng/mL and 0.65 ng/mL, respectively), thus being compliant for the administered drug, but non-compliant for its ketonic metabolite, which is considerably less abundant.

All these irrational conclusions confirm the need of adopting a radically different approach to legally discriminate between exogenous administration of prednisolone and its alleged endogenous origin in cattle. One point that should be taken into account is that the heavy stress condition, supposedly generating endogenous production of prednisolone, usually entails an increase in urinary concentrations of cortisol and cortisone [14-16], whereas the exogenous administration of prednisolone is likely to depress the synthesis and hence the urinary excretion of these endogenous glucocorticoids [28]. In this respect, it is worth noting that in cows we found urinary concentrations of cortisol and cortisone lower than their respective LOQs both before and after prednisolone dosing, while measurable levels of cortisol only could be detected in a few bulls at both T = 0 and T = 33, i.e. prior to treatment and 31 days after the last administration, respectively.

## Conclusion

Several pieces of evidence potentially highlighting the occurrence of prednisolone abuse (e.g. failure to comply with the withdrawal time, masking of an existing pathology, use in lactating cows to speed up the drying off period) may be derived from the urinary excretion profiles emerging from this study involving a typical pharmacological treatment of adult cattle with prednisolone acetate (2 × 0.4-0.5 mg/kg bw i.m. at a 48 h interval). Characteristic features of these profiles are: i) the regular presence of prednisolone and prednisone, the latter usually at lower concentration, even after three to four weeks after the first dosing, ii) the very low cortisol and cortisone levels at any checked timepoint, iii) the consistent occurrence of 20β-dihydroprednisolone and, more sporadically, other reduced or oxidized metabolites of prednisolone and prednisone, and iv) the absence of measurable concentrations (<CCα/LOQ) of prednisolone, prednisone and any other prednisolone metabolite before the treatment. Our findings also indicate that the merely quantitative approach currently adopted (5 ng/mL cut-off concentration for the parent drug - prednisolone - in urine) does not appear to be adequate to ascertain the illicit exposure of cattle to prednisolone according to both a growth-promoting schedule [8] and, as assessed here, also to a therapeutic treatment, at least after 3 to 4 weeks after treatment discontinuation. A more biologically-oriented strategy involving the simultaneous determination of urinary cortisol and cortisone, together with prednisolone, prednisone and at least 20β-dihydroprednisolone among metabolites, is likely to represent a more effective approach for surveillance purposes and consumer’s protection.

## Additional file

Additional file 1: Table S1. Prednisolone (concentration in ng/mL). **Table S2.** Prednisone (concentration in ng/mL). **Table S3.** 20β-dihydroprednisolone (concentration in ng/mL). **Table S4.** 20α-dihydroprednisolone (concentration in ng/mL). **Table S5.** 20β-dihydroprednisone (concentration in ng/mL). **Table S6.** 6β-hydroxyprednisolone (concentration in ng/mL). Urinary concentration values of the analytes for each animal at each sampling time.

## Abbreviations

ESI: Electrospray ionization
CE: Collision energy
CXP: Cell exit potential
LOD: Limit of detection
LOQ: Limit of quantification
CCα: Decision limit
